# Vitamin D3-induced hypercalcemia increases carbon tetrachloride-induced hepatotoxicity through elevated oxidative stress in mice

**DOI:** 10.1371/journal.pone.0176524

**Published:** 2017-04-27

**Authors:** Hiroki Yoshioka, Haruki Usuda, Nobuhiko Miura, Nobuyuki Fukuishi, Tsunemasa Nonogaki, Satomi Onosaka

**Affiliations:** 1 College of Pharmacy, Kinjo Gakuin University, Omori, Moriyamaku, Nagoya, Aichi, Japan; 2 Faculty of Nutrition, Kobe Gakuin University, 518 Arise, Ikawadani-cho, Nishi-ku, Kobe, Hyogo, Japan; 3 Department of Pharmacology, Shimane University Faculty of Medicine, Enya-cho, Izumo, Shimane, Japan; 4 Division of Health Effects Research, Japan National Institute of Occupational Safety and Health, Nagao, Tamaku, Kawasaki, Kanagawa, Japan; University of Navarra School of Medicine and Center for Applied Medical Research (CIMA), SPAIN

## Abstract

The aim of this study was to determine whether calcium potentiates acute carbon tetrachloride (CCl_4_) -induced toxicity. Elevated calcium levels were induced in mice by pre-treatment with cholecalciferol (vitamin D3; V.D3), a compound that has previously been shown to induce hypercalcemia in human and animal models. As seen previously, mice injected with CCl_4_ exhibited increased plasma levels of alanine aminotransferase, aspartate aminotransferase, and creatinine; transient body weight loss; and increased lipid peroxidation along with decreased total antioxidant power, glutathione, ATP, and NADPH. Pre-treatment of these animals with V.D3 caused further elevation of the values of these liver functional markers without altering kidney functional markers; continued weight loss; a lower lethal threshold dose of CCl_4_; and enhanced effects on lipid peroxidation and total antioxidant power. In contrast, exposure to V.D3 alone had no effect on plasma markers of liver or kidney damage or on total antioxidant power or lipid peroxidation. The potentiating effect of V.D3 was positively correlated with elevation of hepatic calcium levels. Furthermore, direct injection of CaCl_2_ also enhanced CCl_4_-induced hepatic injury. Since CaCl_2_ induced hypercalcemia transiently (within 3 h of injection), our results suggest that calcium enhances the CCl_4_-induced hepatotoxicity at an early stage via potentiation of oxidative stress.

## Introduction

Carbon tetrachloride (CCl_4_) is widely used in experimental animal models of liver failure that mimic human hepatic toxicity. The mechanism of CCl_4_ hepatotoxicity has been thoroughly studied since 1967, including the use of *in vivo* models of acute and chronic CCl_4_ poisoning, *ex vivo* perfusion of livers, and the use of isolated or cultured hepatocytes [1, 2]. CCl_4_-induced toxicity is a multifactorial process involving the generation of CCl_4_-derived free radicals [2–5]. The first step is metabolic activation of CCl_4_ by CYP2E1, whereby CCl_4_ is converted to free radicals (trichloromethyl and trichloromethyl peroxy radicals). The second step is binding of these radicals to antioxidant enzymes, including the sulfhydryl (protein thiol) groups of glutathione (GSH). In the third step, these overproduced free radicals increase membrane lipid peroxidation, bind covalently to macromolecules, deplete ATP, and interfere with calcium homeostasis [6–8]. Since sulfhydryl groups are essential elements of the molecular arrangements responsible for the Ca^2+^ transport across cellular membranes, loss of function of these proteins is expected to impair the capacity of microsomes and mitochondria to regulate cellular calcium levels.

Recently, we found that cadmium (Cd) -induced cell cytotoxicity is attenuated by calcium-free medium *in vitro* (unpublished data). These data suggest that calcium is directly involved in Cd-induced toxicity. Because Cd-related toxicity is mediated by GSH depletion, lipid peroxidation, and mitochondrial dysfunction [9–11] (that is, by processes similar to those of CCl_4_-induced toxicity), we hypothesized that calcium might also exacerbate CCl_4_ toxicity.

It is well known that some drugs (e.g., thiazide diuretics) cause hypercalcemia [12, 13]. Treatment with vitamin D commonly has been used to investigate hypercalcemia in animal models [14–16]. In calcium homeostasis, vitamin D3 (V.D3) is a potent serum calcium-raising agent that regulates both calcitonin (CT) and parathyroid hormone (PTH) gene expression [17–19]. Serum calcium is the major secretagogue for CT, a hormone product whose biosynthesis is the main biological activity of thyroid C-cells. Taking advantage of this regulatory mechanism, vitamin D3-induced hypercalcemia has been extensively used.

Therefore, in the current study, we investigated whether hypercalcemic mice exhibited increased CCl_4_-induced toxicity. To examine the effect of calcium on acute CCl_4_ toxicity, we pre-treated animals with V.D3, before determining plasma biochemical markers, hepatic lipid peroxidation, and hepatic calcium levels.

## Material and methods

### Animal treatment

Male ddY mice were purchased from Japan SLC (Hamamatsu, Japan) and were maintained under standard conditions of controlled temperature (24 ± 1°C), humidity (55 ± 5%), and light (12:12 h light/dark cycles) with free access to water and food. Experimental treatments were performed using eight-week-old animals. Following the experiment, any surviving mice were sacrificed using pentobarbital. All experiments were approved by the Institutional Animal Care and Experiment Committee of Kinjo Gakuin University (No. 110).

### Evaluation of the effect of vitamin D3 on CCl_4_ toxicity

**Fig 1:**
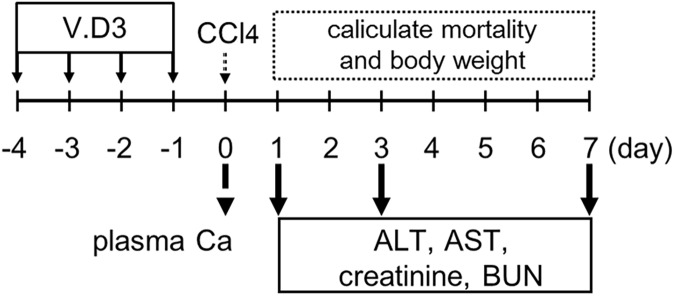
Schematic experimental design of pre-treatment with V.D3 and CCl_4_ injection.

Mice were divided into two groups (olive oil + CCl_4_ and V.D3 + CCl_4_) of twelve mice each. On Days -4 to -1 (i.e., each of the four days prior to CCl_4_ injection), animals were administered once daily (at 24-h intervals) by oral gavage (*per os*; p.o.) with cholecalciferol (vitamin D3; V.D3; Tokyo Chemical Industry, Tokyo, Japan; formulated in olive oil (Nacalai Tesque, Kyoto, Japan)) at 5 mg/kg, or with an equivalent volume of olive oil vehicle alone. On the nominal Day 0 (i.e., twenty-four hours after the final gavage), each mouse was injected intraperitoneally (i.p.) with CCl_4_ (Wako Chemical, Osaka, Japan) at 2 g/kg (5 mL/kg). Before the CCl_4_ injection, we collected pre-dose blood samples from each mouse; these specimens were used to confirm the effects of V.D3 on plasma Ca concentrations. At 24 h after the CCl_4_ injection, three randomly selected mice from each group were euthanized; livers were harvested from each of these animals and flash frozen for storage at -80°C. The remaining mice (nine per group) were maintained on study through Day 7. Once daily following CCl_4_ injection, animals were checked for mortality and body weight was recorded. Additionally, on Days 1, 3, and 7, remaining animals were subjected to blood sampling for determination of blood functional markers. Following the Day-7 procedures, any surviving mice were sacrificed using pentobarbital. Experimental procedure is described in Fig 1.

### Evaluation of role of calcium in CCl_4_ toxicity

Mice were divided into three groups (Ca + olive oil, saline + CCl_4_, and Ca + CCl_4_) of six mice each. Animals were administered i.p. with calcium chloride (CaCl_2_: Wako Chemical; formulated in physiological saline) at 150 mg/kg or with an equivalent volume of saline vehicle. Ten minutes later, animals were administered i.p. with CCl_4_ at 2 g/kg or with an equivalent volume of olive oil. Whole blood was collected at 10 and 30 min and at 1, 3, 6, 12, and 24 h (the last by terminal bleed) after CaCl_2_ injection. At each time point, whole blood specimens were centrifuged (3000× g, 10 min), and the plasma supernatants were frozen and stored at -80°C pending use for determination of plasma calcium concentrations (all time points) or hepatic injury markers (terminal samples). Following the terminal bleeds (at 24 h after i.p. injections), mice of each group were euthanized and livers were harvested. Liver specimens were flash-frozen and stored at -80°C pending use for determination of hepatic calcium levels.

### Plasma biochemical analysis

Plasma calcium levels were measured using the calcium-E test (Wako Chemical) according to the manufacturer’s instructions. Plasma sample (2.5 μL) was mixed with substrate buffer (100 μL) and coloring reagent (50 μL). The absorbance of the reaction mixture was measured at 610 nm.

Plasma alanine aminotransferase (ALT) and aspartate aminotransferase (AST) activities were measured using the Transaminase CII Test Wako (Wako Chemical) according to the manufacturer’s instructions and as previously described [20, 21]. Concentrations of plasma creatinine and blood urea nitrogen (BUN) were measured using Creatinine Liquid Reagents Assay (DIAZYME, Poway, CA) and BUN Wako Test (Wako Chemical), respectively, according to the manufacturer’s instructions and as previously described [22, 23]. For relative quantification, calibration curves were prepared using standard solutions.

### Isolation of total RNA and qRT-PCR assay

Total RNA was extracted from 0.1 g liver sections using the ISOGEN II kit (Nippon Gene, Tokyo, Japan). qRT-PCR was performed with One Step SYBR PrimeScript PLUS RT-PCR kit (Perfect Real Time) (Takara Bio, Shiga, Japan) using an Applied Biosystems 7300 system (Applied Biosystems, Foster City, CA). PCR conditions were as previously described [24]. Primer pairs are shown in Table 1. Relative expression of each mRNA was determined using the standard curve method. The amount of each target mRNA quantified was normalized against that of GAPDH-encoding mRNA.

**Table 1:** Oligonucleotide primer sequences and PCR conditions for real-time RT-PCR.

Gene	Primer sequences	PCR Product
(Accession No.)	Sequence (5’ to 3’)	length (bp)
CYP2E1	Forward CAT TCC TGT GTT CCA GGA GTA CAA G	91
(NM_021282)	Reverse GAT ACT TAG GGA AAA CCT CCG CAC	
GCLC	Forward TAC CAC GCA GTC AAG GAC C	132
(NM_010295)	Reverse AGT CTC AAG AAC ATC GCC TCC	
GCLM	Forward CGG GAA CCT GCT CAA CTG G	117
(NM_008129)	Reverse TCG GGG CTG ATT TGG GAA CTC	
GAPDH	Forward TGG TGA AGG TCG GTG TGA AC	98
(NM_008084)	Reverse GTC GTT GAT GGC AAC AAT CTC C	

### Histopathological findings

For histological analysis, a portion of the left liver lobe from each animal were perfused with 15% phosphate-buffered neutral formalin (pH 7.2), dehydrated, and embedded in paraffin. Embedded tissues were sectioned at 4 μm and stained with hematoxylin and eosin (H&E), Masson trichrome (MT), or von Kossa. MT stain kit and von Kossa stain were purchased from ScyTek Laboratories, Inc. (Logan, UT, USA) and conducted accordance with manufacture’s instructions. Histopathological features were observed using a light microscope.

### Measurement of malondialdehyde levels in the liver

The total malondialdehyde (MDA) levels and total antioxidant power in the liver were examined by colorimetric microplate assay (Oxford Biochemical Research, Oxford, MI) according to the manufacturer's protocol and as previously described [22, 23].

### Determination of glutathione (GSH) levels in the liver

Hepatic GSH levels were measured using GSSG/GSH quantification kit (Dojindo Laboratories, Kumamoto, Japan) according to the manufacturer’s instructions and as previously described [25].

### Measurement of ATP and NADPH levels in the liver

Hepatic ATP and NADPH levels were measured using ATP Colorimetric / Fluorometric Assay kit (BioVision, Inc., Mountain View, CA, USA) and NADH/NADPH Assay kit (BioAssay Systems, Hayward, CA, USA), respectively. These tests were conducted accordance with manufacture’s instructions.

### Determination of liver calcium concentrations

Individual liver specimens (0.2–0.3 g each) were digested in 0.5 mL of concentrated nitric acid in glass test tubes. The temperature was held at 80°C for 1 h, then gradually increased (at 10°C per h) to 130°C. When the acid-digested specimens became transparent, volumes of the digests were raised to 5 mL with distilled water, and calcium concentrations were determined by atomic absorption using a Z-2300 (Hitachi, Tokyo, Japan).

### Statistical analysis

All data from the control and treatment groups were obtained from the same numbers of replicated experiments. All experiments were performed independently at least two times. Two-group comparisons were made using Student’s *t*-test or Welch’s *t-*test; multiple comparisons were analyzed using One-Way ANOVA with post-hoc Tukey-Kramer’s test. Tests were two-tailed. The results of the survival tests were analyzed by means of χ^2^ analysis. All statistical analyses were performed using SPSS 19.0J software (Chicago, IL). Values of *P* < 0.05 were considered statistically significant.

## Results

### Effect of pre-treatment with V.D3 on CCl_4_ acute toxicity, as assessed by body weight and mortality

**Fig 2:**
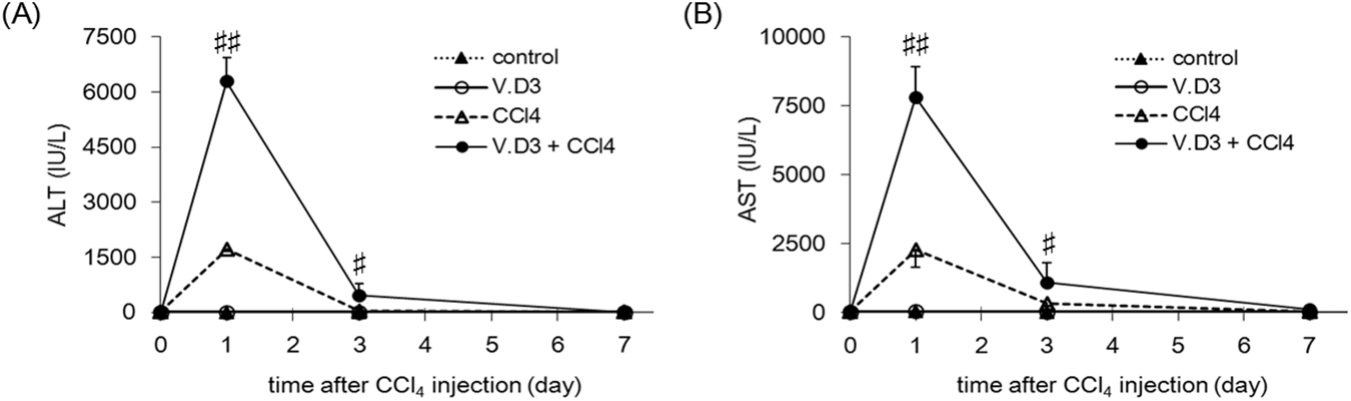
Effect of pre-treatment with V.D3 on CCl_4_ toxicity, as assessed by plasma ALT and AST levels. Mice were pre-treated with olive oil (vehicle) or with V.D3 (at 5 mg/kg) administered as four once-daily p.o. doses. At 24 h after the final pre-treatment, mice of both groups were injected i.p. with CCl_4_ (at 2 g/kg). Plasma ALT (A) and AST (B) activities were determined at 0, 1, 3, and 7 days after CCl_4_ injection. Data are presented as mean ± S.D. of 4–9 mice. ^#^ *P* < 0.05, ^##^ *P* < 0.01 versus CCl_4_ group on the respective day.

**Fig 3:**
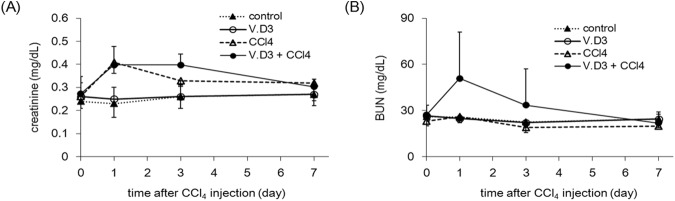
Effect of pre-treatment with V.D3 on CCl_4_ toxicity, as assessed by creatinine and BUN levels. Mice were treated as described in legend for Fig 2. Plasma creatinine (A) and BUN (B) levels were determined at 0, 1, 3, and 7 days after CCl_4_ injection. Data are presented as mean ± S.D. of 4–9 mice.

To determine the effects of V.D3 pre-treatment, we performed analysis of plasma biochemical markers. Four-time, once-daily pre-treatment with V.D3 significantly increased plasma Ca concentrations to 13.0 mg/dL compared to the control value of 7.7 mg/dL (Table 2); these elevated levels would be classified as severe hypercalcemia. In contrast, plasma levels of ALT and AST (markers of hepatic injury; Fig 2) and of creatinine and BUN (markers of kidney injury; Fig 3) were comparable between V.D3- and olive oil-treated groups.

**Table 2:** Effect of pre-treatment with V.D3 on plasma calcium concentrations.

plasma calcium (mg/dL)	
olive oil	7.77 ± 0.70
vitamin D3	13.0 ± 0.97**

Mice were injected p.o. with 5 mg/kg V.D3 four times per 24 h. 24 h after final pre-treatment, plasma calcium concentration was determined. Data indicate mean ± S.D. of nine mice.

**, significantly different from compared values (**P < 0.*01)*.

**Fig 4:**
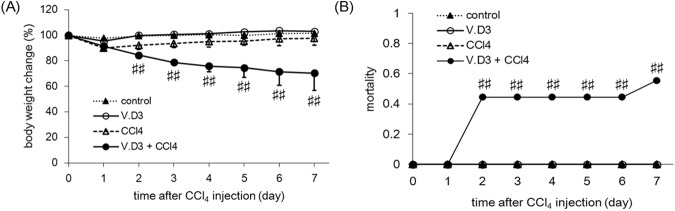
Effect of pre-treatment with V.D3 on CCl_4_ toxicity, as assessed by body weight change and mortality. Mice were treated as described in legend for Fig 2. Body weights (normalized to baseline) (A) and mortality (B) were recorded every 24 h through the 7^th^ day after CCl_4_ injection. Data are presented as mean ± S.D. of 4–9 mice. ^##^ *P* < 0.01 versus CCl_4_ group on the respective day.

These pre-treated animals were administered i.p. with CCl_4_ at 2 g/kg. Animals pre-treated with olive oil (instead of V.D3) and then injected with CCl_4_ exhibited a transient loss of approximately 10% body weight on the first day and subsequent recovery from Day 2 (Fig 4A). In contrast, weight loss in the hypercalcemic mice (pre-treated with V.D3) continued in the days following CCl_4_ injection, achieving approximately 30% loss of weight by Day 7 (compared to baseline), a change that was significant compared to that in the control group. In addition, mortality was significantly elevated in the V.D3 + CCl_4_ treatment group compared to the control animals (Fig 4B). Notably, none of the mice died following CCl_4_ injection, while 55.6% (5 of 9; 4 on Day 2 and 1 on Day 7) of the hypercalcemic mice were found dead in the week following CCl_4_ injection.

### Changes in hepatic and renal injury markers in CCl_4_-exposed mice pre-treated with V.D3

To reveal the target organ of CCl_4_-induced toxicity under hypercalcemic conditions, we next examined hepatic injury markers in the CCl_4_-treated mice. As shown in Fig 2, pre-treatment with V.D3 significantly potentiated the increase in plasma ALT and AST levels seen following CCl_4_ injection; these parameters recovered by the 7^th^ day after CCl_4_ injection.

In parallel with the measurement of ALT and AST, we evaluated plasma creatinine and BUN levels, which are markers of renal injury. As shown in Fig 3A, CCl_4_ exposure yielded significant increases (in both groups) in creatinine levels at Days 1 and 3 (compared to respective baseline values), but these effects did not differ significantly between groups (i.e., for animals pre-treated with V.D3 rather than olive oil). On the other hand, although CCl_4_ exposure yielded an increase (compared to baseline) in Day-1 BUN in animals pre-treated with V.D3, this effect was not significant (at any of the time points) compared to the values obtained with animals pre-treated with olive oil (Fig 3B).

### Effect of pre-treatment with V.D3 on CCl_4_ acute toxicity, as assessed by hepatic CYP2E1 levels

**Fig 5:**
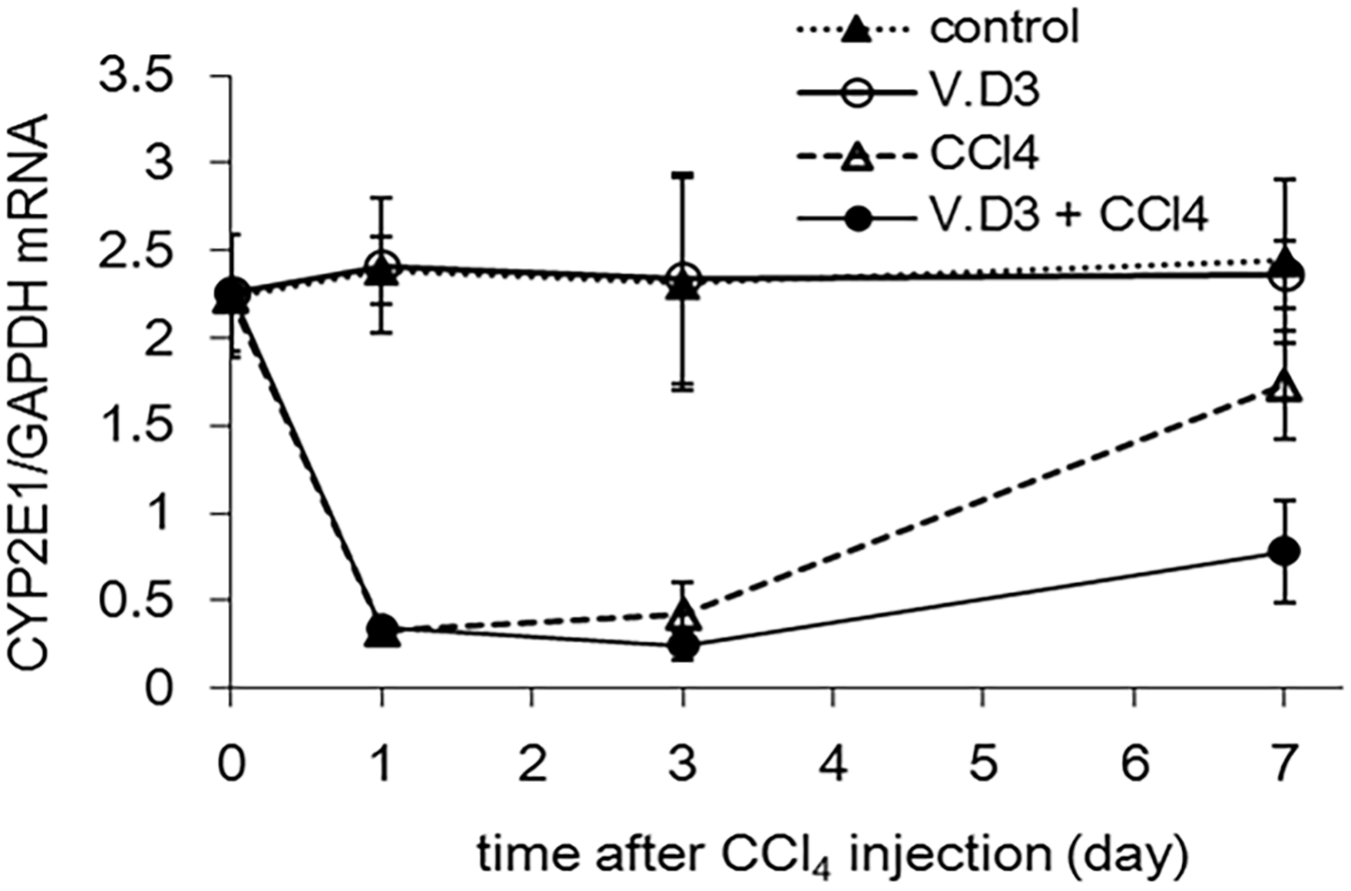
Effect of pre-treatment with V.D3 on CCl_4_ toxicity, as assessed by hepatic CYP2E1 mRNA level. Mice were treated as described in legend for Fig 2. Hepatic CYP2E1 mRNA levels were determined at 0, 1, 3, and 7 days after CCl_4_ injection. Data are presented as mean ± S.D. of 4–9 mice.

In addition to plasma injury markers, we measured hepatic CYP2E1 mRNA levels since CYP2E1 is a major CYP contribution to CCl_4_ activation [26]. As shown in Fig 5, CCl_4_ exposure indicated significant decreases (in both groups) at Days 1 and 3. On the other hand, although CCl_4_ treated group at Day 7 was recovered in CYP2E1, V.D3 + CCl_4_ group was maintained at low level. Moreover, control and VD.3 group at all days were no significant change in CYP2E1 levels.

### Effect of pre-treatment with V.D3 on CCl_4_ acute toxicity, as assessed by MT stain

**Fig 6:**
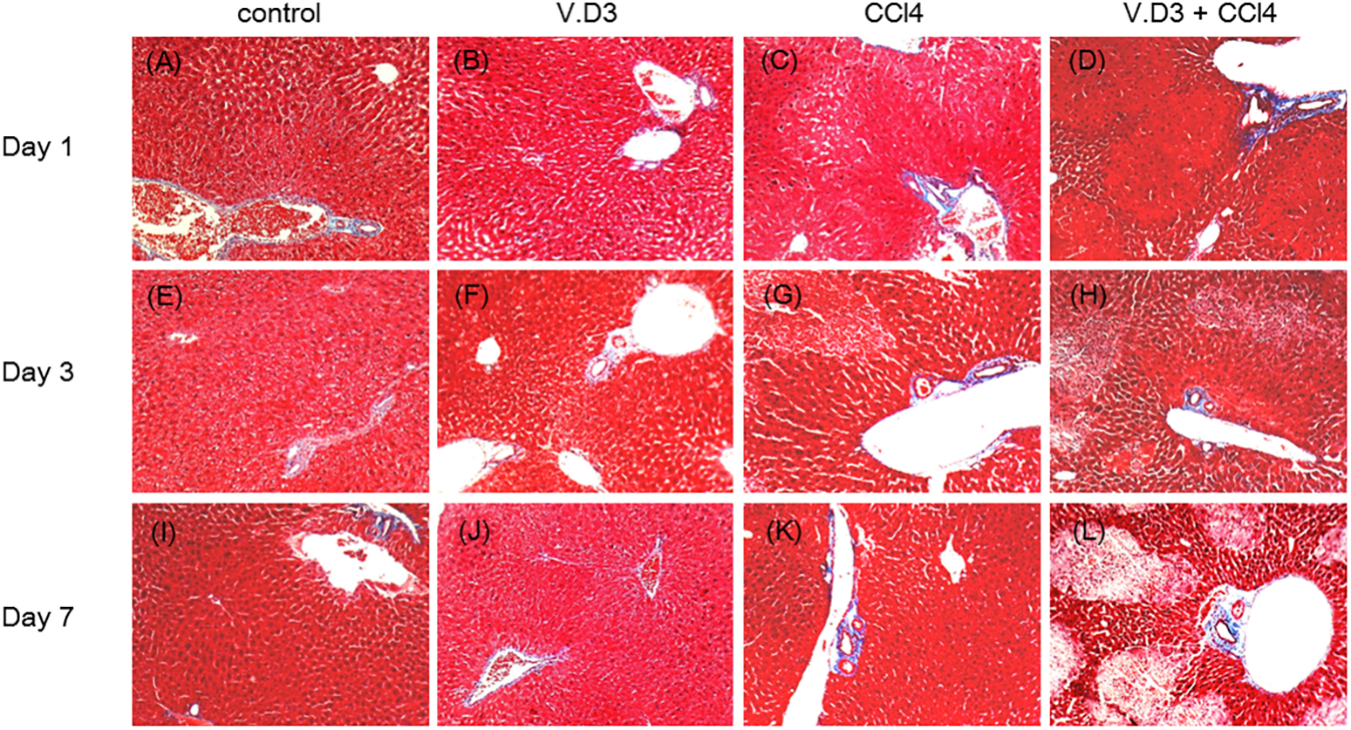
Effect of pre-treatment with V.D3 on CCl_4_ toxicity, as assessed by live fibrosis. Animals were treated as described in legend for Fig 2, and livers were harvested at 24 h, 72 h, or 168 h after CCl_4_ injection. Liver specimens were fixed and stained with MT. Micrographs provide 10× magnified images of representative MT-stained liver sections obtained from the control (A), V.D3 (B), CCl_4_ (C), and V.D3 + CCl_4_ (D) groups at Day 1, control (E), V.D3 (F), CCl_4_ (G), and V.D3 + CCl_4_ (H) groups at Day 3, control (I), V.D3 (J), CCl_4_ (K), and V.D3 + CCl_4_ (L) groups at Day 7, respectivity.

Next, we conducted Masson Trichrome stain since CCl_4_ is well known to induce liver fibrosis [27, 28]. However, hepatic fibrosis was not observed in all groups (Fig 6), suggests generation of hepatic fibrosis need to inject multiple times.

### Changes in morphology, MDA, total antioxidant levels, ATP, and NADPH levels in CCl_4_-exposed mice pre-treated with V.D3

**Fig 7:**
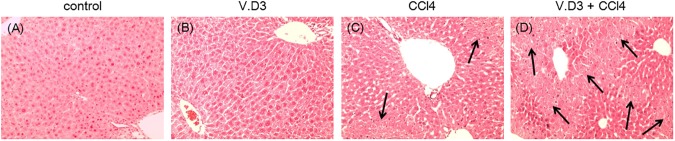
Pretreatment with V.D3 becomes worse animals from acute CCl_4_-induced hepatotoxicity, as assessed by H&E staining. Mice were treated as described in legend for Fig 2. At 24 h after CCl_4_ injection, animals were euthanized and livers were harvested at necropsy. Liver specimens were fixed and processed by standard methods, and sections were stained with H&E (A–D). Micrographs provide 10× magnified images of representative H&E-stained liver sections obtained from the control (A), V.D3 (B), CCl_4_ (C), and V.D3 + CCl_4_ (D) groups. Black arrows indicate area of necrosis.

To further investigate V.D3-induced exacerbation of liver damage, we randomly selected mice from each group, harvested livers from these animals at 24 h after CCl_4_ treatment, and conducted histopathological studies. H&E-stained liver sections from the control and V.D3 groups showed a normal cell morphology and well-preserved cytoplasm, in addition to a clear, plump nucleus (Fig 7A and 7B). In contrast, we observed necrosis in the mice treated with CCl_4_ (Fig 7C). In addition, Pretreatment with V.D3 become exacerbated some, but not all, liver cell necrosis (Fig 7D).

**Fig 8:**
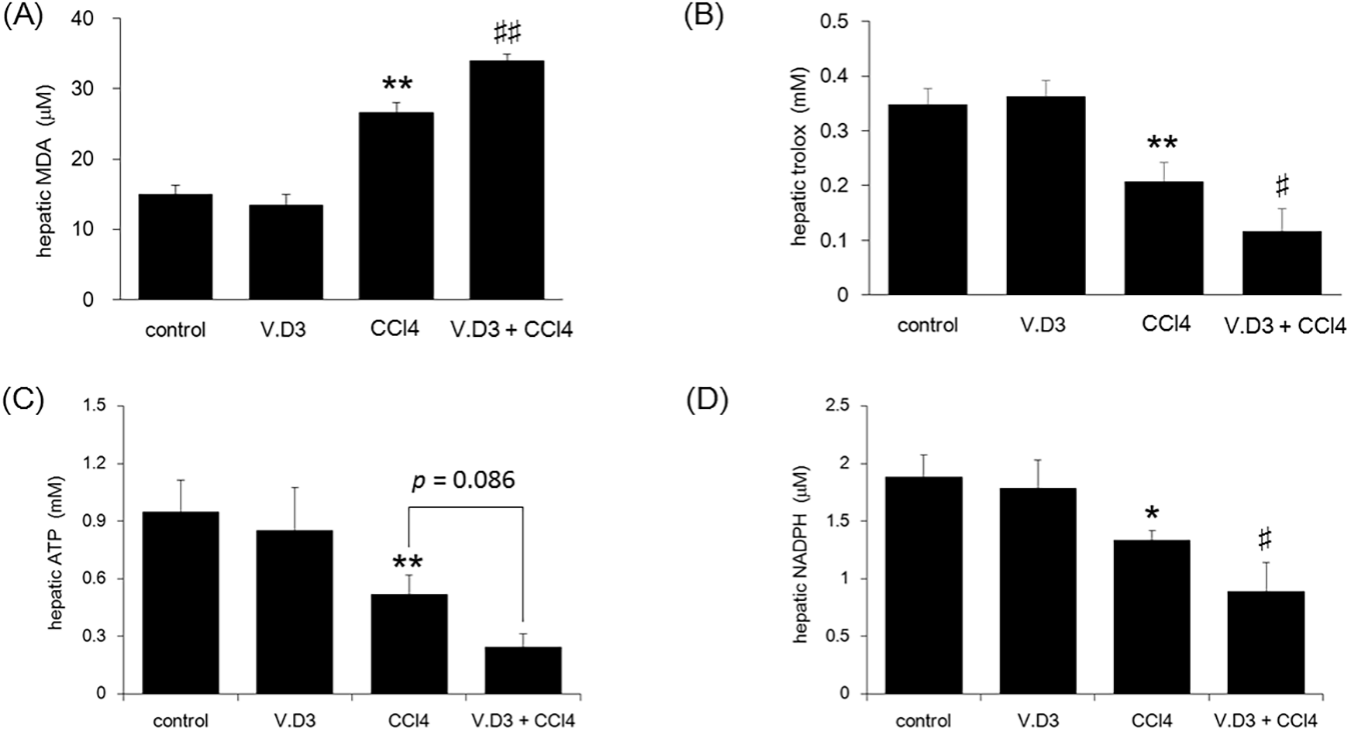
Effect of pre-treatment with V.D3 on CCl_4_ toxicity, as assessed by Day-1 MDA levels, antioxidant power, hepatic ATP levels, and NADPH levels. Mice were treated as described in legend for Fig 2. At 24 h after CCl_4_ injection, animals were euthanized and livers were collected for determination of MDA levels (A), total antioxidant power (B), hepatic ATP levels (C), and hepatic NADPH levels (D). Data are presented as mean ± S.D. of 6 mice. * *P* < 0.05 and ** *P* < 0.01 versus control, ^#^ *P* < 0.05 and ^##^ *P* < 0.01 versus CCl_4_ group.

In parallel with histopathological studies, we measured liver MDA levels as a marker of lipid peroxidation. CCl_4_ treatment significantly increased hepatic MDA levels, both in animals pre-treated with olive oil and in those pre-treated with V.D3 (Fig 8A). Pre-treatment with V.D3 further potentiated the CCl_4_-induced increase in MDA levels (CCl_4_ vs. V.D3 + CCl_4_).

Many studies have suggested that total antioxidant power, ATP, and NADPH can be used as an indicator of oxidative stress. As shown in Fig 8B, CCl_4_-treatment markedly decreased the total antioxidant power, and pre-treatment with V.D3 potentiated the CCl_4_-induced decrease in antioxidant power. Notably, for both hepatic MDA and total oxidant power, values did not differ significantly between animals pre-treated with vehicle and with V.D3. This observation demonstrated that hypercalcemia itself does not induce either of these parameters. In addition, hepatic ATP and NAPDH levels were consistent with total antioxidant power (Fig 8C and 8D).

**Fig 9:**
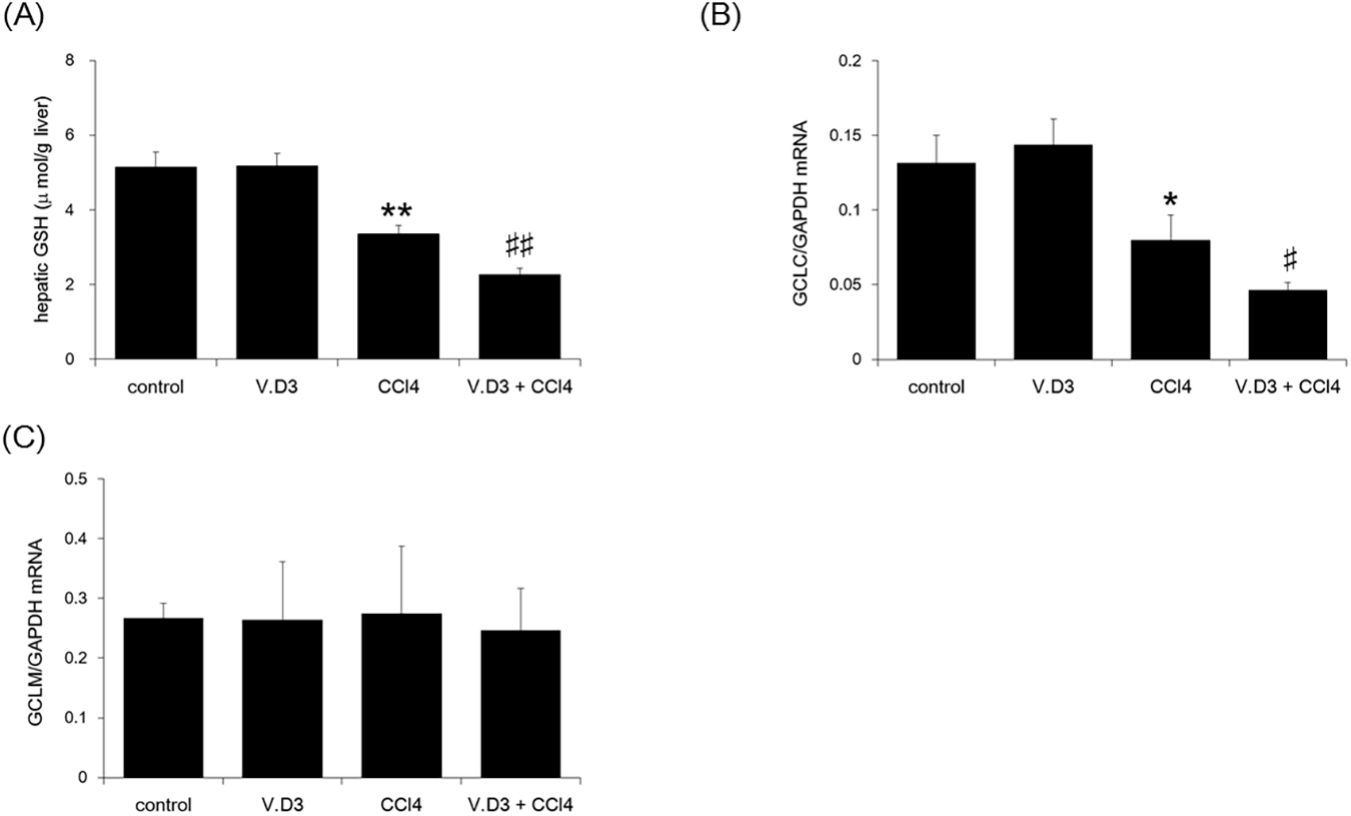
Effect of pre-treatment with V.D3 on CCl_4_ toxicity, as assessed by Day-1 hepatic GSH levels, GCLC, and GCLM levels. Mice were treated as described in legend for Fig 2. At 24 h after CCl_4_ injection, animals were euthanized and livers were collected for determination of GSH levels (A), GCLC mRNA (B), and GCLM mRNA (C). Data are presented as mean ± S.D. of 6 mice. * *P* < 0.05 and ** *P* < 0.01 versus control, ^#^ *P* < 0.05 and ^##^ *P* < 0.01 versus CCl_4_ group.

Moreover, we determined hepatic GSH levels, that is well known to deplete on CCl_4_ administration [29–32]. As shown in Fig 9A, CCl_4_ treatment significantly decreased hepatic GSH levels, both in animals pre-treated with olive oil and in those pre-treated with V.D3. Pre-treatment with V.D3 further potentiated the CCl_4_-induced decrease in GSH levels (CCl_4_ vs. V.D3 + CCl_4_). Moreover, we determined glutamate cysteine ligase catalytic subunit (GCLC) and glutamate cysteine ligase modifier subunit (GCLM) by qRT-PCR assay (Fig 9B and 9C). Although GCLC was same tendency compared with GSH, GCLM was no significant change in all groups in the present study.

### Influence of V.D3 on CCl_4_ acute toxicity as assessed by hepatic calcium levels and calcium stain

**Fig 10:**
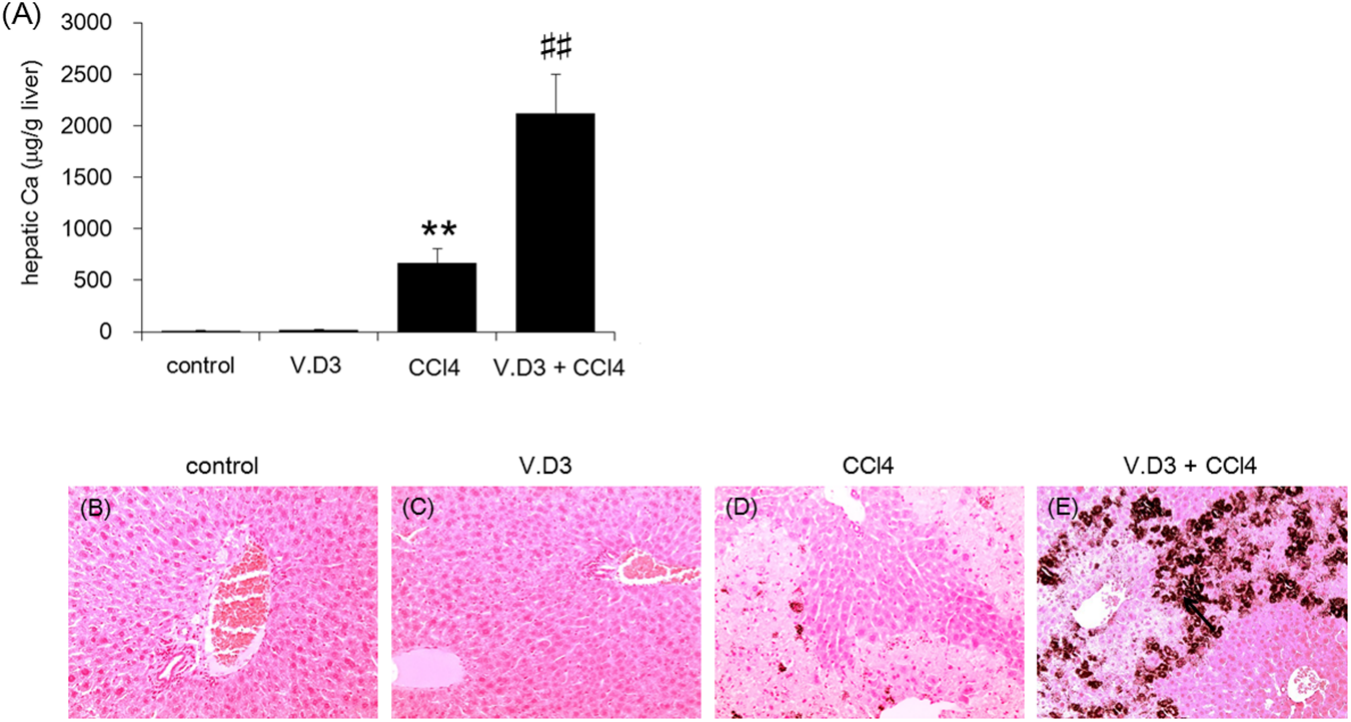
Effect of pre-treatment with V.D3 on CCl_4_ toxicity, as assessed by hepatic calcium levels and calcium stain. Animals were treated as described in legend for Fig 2, and livers were harvested at 24 h after CCl_4_ injection. (A): Hepatic calcium levels at 24 h were determined by atomic absorption spectrometry. Data are presented as mean ± S.D. of 6 mice. * *P* < 0.05 and ** *P* < 0.01 versus control, ^##^ *P* < 0.01 versus CCl_4_ group. (B–E): Liver specimens were fixed and stained with von Kossa. Micrographs provide 10× magnified images of representative von Kossa-stained liver sections obtained from the control (B), V.D3 (C), CCl_4_ (D), and V.D3 + CCl_4_ (E) groups.

As we showed above, pre-treatment with V.D3 yielded increased plasma Ca levels. We next examined whether V.D3 pre-treatment, with or without CCl_4_ exposure, also altered hepatic calcium levels at 24 h post CCl_4_ injection, which we assessed by atomic absorption spectrometry (Fig 10A). In animals pre-treated with olive oil, CCl_4_ injection yielded a significant, 60-fold increase in liver Ca levels. Injection of CCl_4_ in mice pre-treated with V.D3 yielded a further >3-fold elevation in hepatic Ca levels. Notably, pre-treatment with V.D3 yielded a small (1.8-fold) and non-significant increase in hepatic Ca levels compared to pre-treatment with olive oil (in the absence of CCl_4_ injection). This observation demonstrated that V.D3 alone does not induce appreciable hypercalcemia of the liver. In further to investigate Ca involvement, we stained hepatic Ca by von Kossa method. In control and V.D3 groups, Ca deposition was not observed (Fig 10B and 10C). In contrast, Injection of CCl_4_ in mice was slightly confirmed von Kossa positive staining in the area necrosis is not observed (Fig 10D). Moreover, maximum von Kossa staining was confirmed in V.D3 + CCl_4_ group (Fig 10E).

### Direct assessment of Ca effect on CCl_4_ acute toxicity

**Fig 11:**
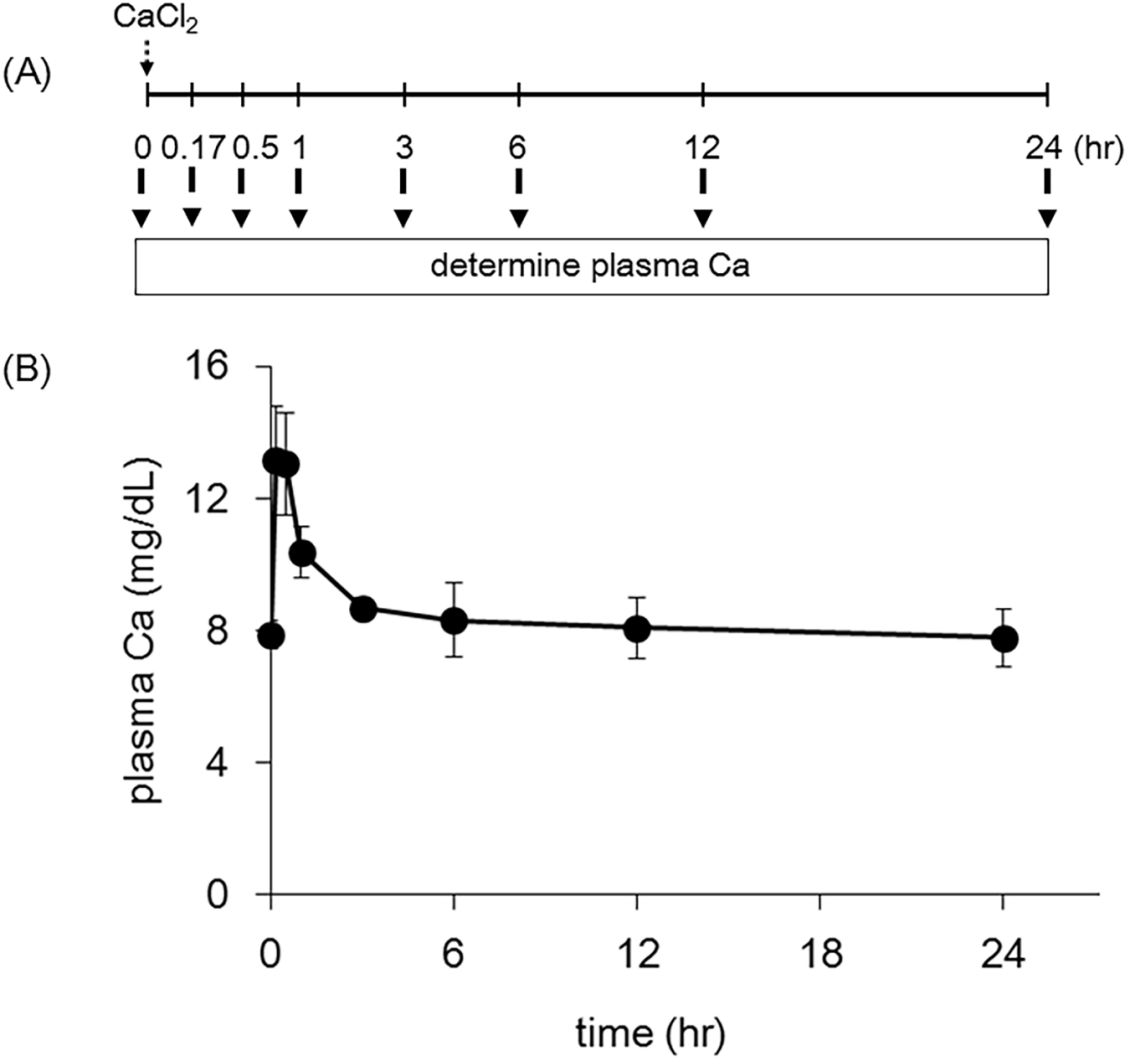
Effect of intraperitoneal injection with CaCl_2_ on plasma calcium levels. Mice were injected i.p. with CaCl_2_ at 150 mg/kg. Plasma calcium levels were determined after 10 and 30 min and at 1, 3, 6, 12, and 24 h after CaCl_2_ injection. (A) and (B) show the schematic experimental design of CaCl_2_ injection and the results, respectively. Data are presented as mean ± S.D. of 6 mice.

In order to confirm the involvement of calcium in CCl_4_ toxicity, we induced hypercalcemia by direct injection of CaCl_2_ and monitored plasma calcium levels for the subsequent 24 h, both with and without concomitant CCl_4_ exposure. As shown in Fig 11B, i.p. injection of CaCl_2_ induced transient (within 3 h) hypercalcemia. When mice with this evanescent hypercalcemia were injected with CCl_4_ (Ca + CCl_4_), the animals exhibited significantly elevated plasma ALT and AST levels and hepatic calcium levels compared to normal-calcemic mice (CCl_4_) (Table 3).

**Table 3:** Effect of pre-treatment with calcium on various parameters associated with CCl_4_-induced acute hepatotoxicity.

	ALT (IU/L)	AST (IU/L)	hepatic Ca (μg/g liver)
Ca	8.69 ± 0.97	46.25 ±19.78	16.49 ± 4.06
CCl_4_	2115 ± 416**	2565 ± 534**	532 ±125**
Ca + CCl_4_	4153 ± 1252^##^	4650 ±767^##^	781 ± 54.0^##^

Mice were injected i.p. with CaCl_2_ (at 150 mg/kg) 10 min before i.p. injection with CCl_4_ (at 2 g/kg). Post 24 h after CCl_4_ injection, plasma ALT, AST and hepatic Ca was measured. Data indicate mean ± S.D. of four or six mice.

**, significantly different from Ca + olive oil group (**P < 0.*01) and*

^##^, significantly different from saline + CCl_4_ group (^##^*P < 0*.*01)*.

## Discussion

The present study demonstrated that pre-treatment with V.D3 potentiated CCl_4_-induced hepatotoxicity and enhanced mouse mortality, without increasing renal toxicity and generation of liver fibrosis. Our previous investigation demonstrated that single i.p. injection of mice with a fatal dose of CCl_4_ (4 g/kg) induced severe hepatotoxicity and moderate renal toxicity [20, 22, 24]; however, the critical target organ that led to mouse death following CCl_4_ injection was not defined. In the current study, V.D3 potentiation of toxicity was observed only in the liver, as indicated by plasma levels of ALT and AST, biochemical markers of hepatic damage. Although pre-treatment with V.D3 significantly increased renal calcium levels compared to those in animals pre-treated with olive oil, renal calcium content did not differ significantly between mice treated with olive oil + CCl_4_ and those treated with V.D3 + CCl_4_ (data not shown). Together, these data suggest that the liver is the primary target organ of acute CCl_4_ toxicity.

CCl_4_ is metabolized and activated by multiple CYPs, including CYP2E1, CYP2B1, and CYP2B2 [2]. In particular, CYP2E1 is a major CYP contribution to CCl_4_ activation [26]. Several literatures reported pre-treatment with phenobarbital, acarbose, or natural products (such as *Salvia officinalis*) have been shown to potentiate the CYP2E1-mediated hepatotoxicity of CCl_4_ [33–36]. Although vitamin D is known to induce the expression of CYP3A and CYP2B6 via activation of the vitamin D receptor (VDR), the pregnane X receptor (PXR), and/or the constitutive androstane receptor (CAR) [37–39], we are not aware of any reports of V.D3-induced expression of CYP2E1, 2B1, or 2B2. In fact, hepatic CYP2E1 expression level was not changed by pretreatment with V.D3. Taken together, these observations indicate that CYPs are not primary mediators of the V.D3 potentiation of CCl_4_ toxicity.

Several studies suggest that a possible molecular mechanism involved in CCl_4_ hepatotoxicity is the disruption of the delicate oxidant/antioxidant balance, which can lead to liver injury via oxidative damage [2, 40]. Our results suggest that V.D3 (or a V.D3-induced factor) triggers an enhancement of CCl_4_-induced toxicity. Since V.D3 has no ability to change every parameters such as antioxidant power, MDA levels, ATP levels, NADPH levels, GSH levels, and GCL subunit levels, V.D3 itself is not an oxidant. We hypothesize that calcium is likely the aggravating factor, given that pre-treatment with CaCl_2_ yielded potentiation of CCl_4_ toxicity similar to that seen with pre-treatment with V.D3, a compound known to induce hypercalcemia. In addition, the extracellular plasma calcium concentration is tightly controlled by a complex homeostatic mechanism involving fluxes of calcium between the extracellular fluid and the kidneys, bones, and hormones. It has been reported that CCl_4_ disrupts hepatic calcium homeostasis [41, 42]. In the current study, CCl_4_-induced hepatic calcium levels were increased by pre-treatment with V.D3, indicating that calcium is a candidate aggravating factor of CCl_4_ toxicity. Moreover, multiple researchers have reported that CCl_4_ significantly decreases the total content of reduced GSH, and that CCl_4_-derived radicals can react with sulfhydryl groups of GSH and other protein thiols [29–32]. Our data also supports these reports since GSH was depleted by CCl_4_ and these depletion levels got worse by pretreatment with V.D3. In addition, GSH is sequentially synthesize catalytic subunit d from glutamate, cysteine, and glycine, which is mainly controlled by GCL. GCL is composed of two subunits, the GCLC and the modifier subunit GCLM. Our study indicated that GCLC was same tendency compared with GSH. In contrast, GCLM was no significant change in all groups in the present study. These data suggests that single injection of CCl_4_ might attack GCLC rather than GCLM since multiple injection of CCl_4_ reduces both parameters [43].

Since some protein thiols are essential components of the molecular rearrangements that are required for Ca^2+^ transport across cell membranes, loss of such thiols may affect the calcium sequestration activity of subcellular compartments; mitochondria and microsomes employ this sequestration to regulate cytosolic calcium levels. Hence, pre-treatment with V.D3 might induce the collapse of these cellular functions by disrupting calcium homeostasis in the cell.

We demonstrated that both V.D3-induced hypercalcemia and direct injection of calcium itself potentiate CCl_4_-induced toxicity; these results suggest that calcium potentiates hepatotoxicity. In addition, we speculate that calcium augments the CCl_4_-induced toxicity within several hours after CCl_4_-injection, given that transient hypercalcemia was observed at the earliest time points following CaCl_2_ injection [44]. It has been reported that CCl_4_-induced hepatotoxicity occurs within 3 h of exposure [45], consistent with our speculation.

In conclusion, we demonstrated that V.D3-induced hypercalcemia or pre-treatment with CaCl_2_ enhances CCl_4_-induced hepatotoxicity, presumably via disruption of calcium homeostasis. To our knowledge, this is the first evidence that calcium enhances CCl_4_-induced hepatotoxicity in the early stage in mice. These findings may have relevance to the mechanism of toxicity of other hepatotoxic compounds.

## Abbreviations

ALT: alanine aminotransferase
AST: aspartate amino transferase
CCl_4_: carbon tetrachloride
V.D3: vitamin D3
ATP: adenosine triphosphate
NADPH: reduced nicotinamide adenine dinucleotide phosphate
